# Supplementary material for: A UMLS-based spell checker for natural language processing in vaccine safety
Source: BMC Med Inform Decis Mak. 2007 Feb 12;7:3. doi: 10.1186/1472-6947-7-3 (PMC1805499; doi:10.1186/1472-6947-7-3)
Supplement: Additional file 13 — RAP application directory. Sets up directory for RAP (RDF application for PHP) source code [file 1472-6947-7-3-S13.gz › rap/doc/phpdoc/model/_constants_php.html]

xml version="1.0" encoding="iso-8859-1"?


Docs for page constants.php


## /constants.php

Description

Description |
Constants

Constants

Description |
Constants

ADODB\_DB\_DRIVER = 'ODBC'
(line 286)

ADODB\_DB\_HOST = 'rap'
(line 287)

ADODB\_DB\_NAME = ''
(line 288)

ADODB\_DB\_PASSWORD = ''
(line 290)

ADODB\_DB\_USER = ''
(line 289)

ADODB\_DEBUG\_MODE = '0'
(line 291)

BNODE\_PREFIX = 'bNode'
(line 63)

CREATE\_MODEL\_WITHOUT\_DUPLICATES = FALSE
(line 103)

DATATYPE\_SHORTCUT\_PREFIX = 'datatype:'
(line 264)

DBMODEL = 'DbModel'
(line 82)

DEFAULT\_ALGORITHM = 'MD5'
(line 39)

DEFAULT\_ENCODING = 'UTF-8'
(line 40)

FIX\_BLANKNODES = true
(line 107)

GENERAL\_PREFIX\_BASE = 'ns'
(line 173)

HIDE\_ADVERTISE = FALSE
(line 139)

HTML\_TABLE\_BNODE\_COLOR = '#FFCCFF'
(line 184)

HTML\_TABLE\_HEADER\_COLOR = '#FFFFFF'
(line 181)

HTML\_TABLE\_LITERAL\_COLOR = '#E7E7EF'
(line 183)

HTML\_TABLE\_RDF\_NS\_COLOR = '#CCFFCC'
(line 185)

HTML\_TABLE\_RESOURCE\_COLOR = '#FFFFCC'
(line 182)

INDENTATION = ' '
(line 41)

INDEX\_TYPE = IND\_DEF
(line 75)

IND\_DEF =
(line 71)

IND\_SO = 3
(line 74)

IND\_SP = 2
(line 73)

IND\_SPO = 1
(line 72)

INFMODELB = 'InfModelB'
(line 84)

INFMODELF = 'InfModelF'
(line 83)

INF\_RES\_DOMAIN = true
(line 328)

INF\_RES\_OWL\_INVERSEOF = true
(line 332)

INF\_RES\_OWL\_SAMEAS = true
(line 330)

INF\_RES\_RANGE = true
(line 326)

INF\_RES\_RULE\_RDFS6 = false
(line 337)

INF\_RES\_RULE\_RDFS8 = false
(line 338)

INF\_RES\_RULE\_RDFS10 = false
(line 339)

INF\_RES\_RULE\_RDFS12 = false
(line 336)

INF\_RES\_RULE\_RDFS13 = false
(line 340)

INF\_RES\_SUBCLASSOF = true
(line 322)

INF\_RES\_SUBPROPERTYOF = true
(line 324)

IN\_DESCRIPTION = 2
(line 113)

IN\_PROPERTY\_EMPTY\_RESOURCE = 5
(line 116)

IN\_PROPERTY\_LITERAL = 6
(line 117)

IN\_PROPERTY\_PARSE\_TYPE\_COLLECTION = 11
(line 122)

IN\_PROPERTY\_PARSE\_TYPE\_LITERAL = 7
(line 118)

IN\_PROPERTY\_PARSE\_TYPE\_RESOURCE = 8
(line 119)

IN\_PROPERTY\_RESOURCE = 4
(line 115)

IN\_PROPERTY\_UNKNOWN\_OBJECT = 3
(line 114)

IN\_RDF = 1
(line 112)

IN\_TOP\_LEVEL =
(line 111)

IN\_UNKNOWN = 10
(line 121)

IN\_XML = 9
(line 120)

LINEFEED = chr(10)
(line 42)

MAX\_ALLOWED\_ABBREVIATED\_LENGTH = 60
(line 174)

MEMMODEL = 'MemModel'
(line 81)

N3SER\_BNODE\_SHORT = TRUE
(line 166)

NAMESPACE\_SEPARATOR\_CHAR = '^'
(line 109)

NAMESPACE\_SEPARATOR\_STRING = '^'
(line 110)

NO\_INDEX = -1
(line 70)

ONTMODEL = 'OntModel'
(line 85)

OWL\_INVERSE\_OF = 'inverseOf'
(line 253)

OWL\_PREFIX = 'owl'
(line 251)

OWL\_SAME\_AS = 'sameAs'
(line 252)

OWL\_URI = 'http://www.w3.org/2002/07/owl#'
(line 250)

PACKAGE\_DBASE = 'model/DBase.php'
(line 49)

PACKAGE\_INFMODEL = 'infModel/InfModelP.php'
(line 54)

PACKAGE\_MODEL = 'model/ModelP.php'
(line 47)

PACKAGE\_ONTMODEL = 'ontModel/OntModelP.php'
(line 56)

PACKAGE\_RDQL = 'rdql/RDQL.php'
(line 53)

PACKAGE\_RESMODEL = 'resModel/ResModelP.php'
(line 55)

PACKAGE\_SYNTAX\_N3 = 'syntax/SyntaxN3.php'
(line 51)

PACKAGE\_SYNTAX\_RDF = 'syntax/SyntaxRDF.php'
(line 50)

PACKAGE\_UTILITY = 'util/Utility.php'
(line 48)

PACKAGE\_VOCABULARY = 'vocabulary/Vocabulary.php'
(line 52)

RDFAPI\_ERROR = 'RDFAPI error '
(line 38)

RDFS\_CLASS = 'Class'
(line 236)

RDFS\_COMMENT = 'comment'
(line 243)

RDFS\_DATATYPE = 'Datatype'
(line 238)

RDFS\_DOMAIN = 'domain'
(line 235)

RDFS\_IS\_DEFINED\_BY = 'isDefinedBy'
(line 241)

RDFS\_LABEL = 'label'
(line 242)

RDFS\_LITERAL = 'Literal'
(line 239)

RDFS\_RANGE = 'range'
(line 234)

RDFS\_RESOURCE = 'Resource'
(line 237)

RDFS\_SEE\_ALSO = 'seeAlso'
(line 240)

RDFS\_SUBCLASSOF = 'subClassOf'
(line 232)

RDFS\_SUBPROPERTYOF = 'subPropertyOf'
(line 233)

RDFS\_VOCABULARY = 'RdfsVocabulary.php'
(line 87)

RDF\_ABOUT = 'about'
(line 196)

RDF\_ABOUT\_EACH = 'aboutEach'
(line 197)

RDF\_ABOUT\_EACH\_PREFIX = 'aboutEachPrefix'
(line 198)

RDF\_ALT = 'Alt'
(line 209)

RDF\_BAG = 'Bag'
(line 207)

RDF\_BAG\_ID = 'bagID'
(line 199)

RDF\_DATATYPE = 'datatype'
(line 216)

RDF\_DATATYPE\_SCHEMA\_URI = 'http://www.w3.org/TR/xmlschema-2'
(line 230)

RDF\_DESCRIPTION = 'Description'
(line 194)

RDF\_FIRST = 'first'
(line 222)

RDF\_ID = 'ID'
(line 195)

RDF\_LI = 'li'
(line 210)

RDF\_LIST = 'List'
(line 219)

RDF\_NAMESPACE\_PREFIX = 'rdf'
(line 192)

RDF\_NAMESPACE\_URI = 'http://www.w3.org/1999/02/22-rdf-syntax-ns#'
(line 191)

RDF\_NIL = 'nil'
(line 220)

RDF\_NODEID = 'nodeID'
(line 215)

RDF\_OBJECT = 'object'
(line 214)

RDF\_OBJECT\_TYPE\_BNODE = 3
(line 131)

RDF\_OBJECT\_TYPE\_LITERAL = 1
(line 129)

RDF\_OBJECT\_TYPE\_RESOURCE =
(line 128)

RDF\_OBJECT\_TYPE\_XML = 2
(line 130)

RDF\_PARSE\_TYPE = 'parseType'
(line 202)

RDF\_PARSE\_TYPE\_COLLECTION = 'Collection'
(line 205)

RDF\_PARSE\_TYPE\_LITERAL = 'Literal'
(line 203)

RDF\_PARSE\_TYPE\_RESOURCE = 'Resource'
(line 204)

RDF\_PREDICATE = 'predicate'
(line 213)

RDF\_PROPERTY = 'Property'
(line 218)

RDF\_RDF = 'RDF'
(line 193)

RDF\_RESOURCE = 'resource'
(line 200)

RDF\_REST = 'rest'
(line 221)

RDF\_SCHEMA\_PREFIX = 'rdfs'
(line 231)

RDF\_SCHEMA\_URI = 'http://www.w3.org/2000/01/rdf-schema#'
(line 229)

RDF\_SEEALSO = 'seeAlso'
(line 217)

RDF\_SEQ = 'Seq'
(line 208)

RDF\_STATEMENT = 'Statement'
(line 211)

RDF\_SUBJECT = 'subject'
(line 212)

RDF\_SUBJECT\_TYPE\_ANONYMOUS = 3
(line 126)

RDF\_SUBJECT\_TYPE\_BNODE = 4
(line 127)

RDF\_SUBJECT\_TYPE\_DISTRIBUTED = 1
(line 124)

RDF\_SUBJECT\_TYPE\_PREFIX = 2
(line 125)

RDF\_SUBJECT\_TYPE\_URI =
(line 123)

RDF\_TYPE = 'type'
(line 206)

RDF\_VALUE = 'value'
(line 201)

RDF\_XMLLITERAL = 'XMLLiteral'
(line 223)

RDQL\_AND\_ERR = RDQL\_ERR.'in the AND clause: '
(line 303)

RDQL\_ERR = 'RDQL error '
(line 298)

RDQL\_SEL\_ERR = RDQL\_ERR.'in the SELECT clause: '
(line 300)

RDQL\_SRC\_ERR = RDQL\_ERR.'in the SOURCE clause: '
(line 301)

RDQL\_SYN\_ERR = 'RDQL syntax error '
(line 299)

RDQL\_USG\_ERR = RDQL\_ERR.'in the USING clause: '
(line 304)

RDQL\_WHR\_ERR = RDQL\_ERR.'in the WHERE clause: '
(line 302)

RESMODEL = 'ResModel'
(line 86)

SER\_RDF\_QNAMES = TRUE
(line 158)

SER\_SORT\_MODEL = TRUE
(line 150)

SER\_USE\_ATTRIBUTES = FALSE
(line 146)

SER\_USE\_ENTITIES = FALSE
(line 142)

SER\_XML\_DECLARATION = TRUE
(line 162)

UNIC\_RDF = TRUE
(line 99)

USE\_ANY\_QUOTE = FALSE
(line 172)

USE\_CDATA = FALSE
(line 170)

VALIDATE\_IDS = TRUE
(line 95)

XML\_LANG = 'lang'
(line 263)

XML\_NAMESPACE\_DECLARATION\_PREFIX = 'xmlns'
(line 261)

XML\_NAMESPACE\_PREFIX = 'xml'
(line 260)

XML\_NAMESPACE\_URI = 'http://www.w3.org/XML/1998/namespace'
(line 262)

Documentation generated on Wed, 20 Oct 2004 13:59:12 +0200 by phpDocumentor 1.3.0RC3
